# Metal pattern-based planar sub-THz filter in coplanar waveguide on optically transparent substrate

**DOI:** 10.1038/s41598-025-15178-3

**Published:** 2025-09-26

**Authors:** Jaroslav Havlíček, Daniel Havelka, Michal Cifra

**Affiliations:** Institute of Photonics and Electronics of the Czech Academy of Sciences, Prague, Czechia

**Keywords:** Electronics, photonics and device physics, Engineering

## Abstract

The sub-terahertz and terahertz frequency bands (0.1–10 THz) hold immense potential for revolutionizing diverse fields, ranging from advanced telecommunications to biomedical diagnostics and structural biology. Despite the rapidly growing interest in these frequencies, there is a significant gap in the development of compact, high-performance sub-terahertz devices on optically transparent substrates. Such substrates are crucial for integration with optical microscopy and spectroscopy techniques, enabling applications in biophysics and material sciences. To address this technology gap, this paper presents the design, fabrication, and measurement of a compact band-stop filter unit operating in the sub-terahertz F-band (90–140 GHz) on an optically transparent substrate. The unit utilizes a $\lambda$/4 open-end folded stub resonator structure integrated within a coplanar waveguide. Different coupling variants between the stub and coplanar waveguide were analyzed using frequency domain simulations. The device is compatible with advanced optical microscopy techniques since it is implemented on an optically transparent quartz glass substrate. This feature enables future applications in spectroscopy or biophysics thanks to straightforward integrability with microfluidics. The unit can be cascaded into a higher-order filter to enhance its performance.

**Supplementary Information:**

The online version contains supplementary material available at 10.1038/s41598-025-15178-3.

## Introduction

The sub-terahertz (subTHz) and terahertz (THz) bands (0.1-10 THz)^1^ present significant opportunities across a wide range of industrial and research fields^2^. In this paper, we contribute to these efforts by providing a specialised new type of passive component for subTHz systems.

Thanks to their increased bandwidth, subTHz and THz frequencies are poised to revolutionize connectivity in the 6G era by enabling unprecedented data rates^3,4^. Beyond telecommunication, astronomical observations greatly benefit from subTHz and THz radiation, as these frequencies allow scientists to peer into cold, dusty regions of space, revealing crucial insights into star formation and the structure of galaxies^5^. In the realm of physical sciences, THz waves serve as a vital tool for investigating quantum effects at microscopic scales in condensed matter physics^5^. Likewise, they play an important role in spintronics, helping to explore ultrafast magnetic dynamics and energy transfer processes^2^. Security applications also leverage technology, with its high sensitivity enabling the detection of concealed objects, such as weapons and explosives, as well as the inspection of structural defects in various materials^2^. In the field of analytical chemistry, (sub)THz spectroscopy is advancing drug development and quality control, offering enhanced capabilities for studying drug crystallinity, molecular interactions, and pharmaceutical coatings^6,7^. Material sciences, especially polymer research, benefit from techniques for analyzing polymer morphology, glass transitions, and phase separations, providing deeper insights into material properties^8^. Agriculture and food science are no exception to these advancements, with (sub)THz technology being utilized for non-destructive testing, monitoring water content, detecting contaminants, and assessing food drying processes^9,10^. In the biomedical domain, (sub)THz technology is unlocking new potential in non-invasive diagnostics, biosensing, and biochips. Its high penetration and non-ionizing properties make it particularly valuable for cancer detection, tissue characterization, and medical imaging applications^7,11^. Lastly, in structural biology and biophysics, (sub)THz waves shed light on the dynamics of biomolecules, especially proteins. These essential molecules exhibit global vibration modes within the subTHz range, with each vibration tied to dipole moment variations. This enables proteins to be studied as electrically small antennas, offering new perspectives on their functional mechanisms and structural changes linked to diseases^12–16^.

While there are existing subTHz-THz dielectric devices fabricated from silicon^17–20^, which is optically opaque, many of these specialized applications demand THz devices fabricated on optically transparent substrates so that they can be interrogated with optical spectroscopy or transmission microscopy techniques. Yet demonstrations of microwave devices above 100 GHz on such substrates remain scarce; most reported implementations operate only at lower frequencies, typically from a few gigahertz up to a few tens of gigahertz^21–25^. That is also a case of our previous work in this field^26,27^. In the range of THz bands usually a direct beam is used instead of a waveguide^28,29^ or in case of transmission line-based sensing, only simple device geometries are used^30–34^. To fully harness the potential of these diverse subTHz-THz applications outlined above, resonator devices are essential for frequency selection, spectral signal filtering, sensing, and efficient integration within subTHz and THz systems, enabling tailored performance across telecommunications, spectroscopy, biophysics, and material analysis.

This paper focuses on design, fabrication and experimental demonstration of such resonator structure in the role of a band-stop filter unit for the F-band (90-140 GHz). The proposed filter unit design emphasizes electrically small size, compactness, and manufacturing simplicity. These characteristics are important for the cheap mass production of devices required by the above-mentioned subTHz-THz and 5G/6G applications. For manufacturing simplicity, a compact filter topology should ideally be based on a single-layer (uniplanar) transmission line.

**Fig. 1:**
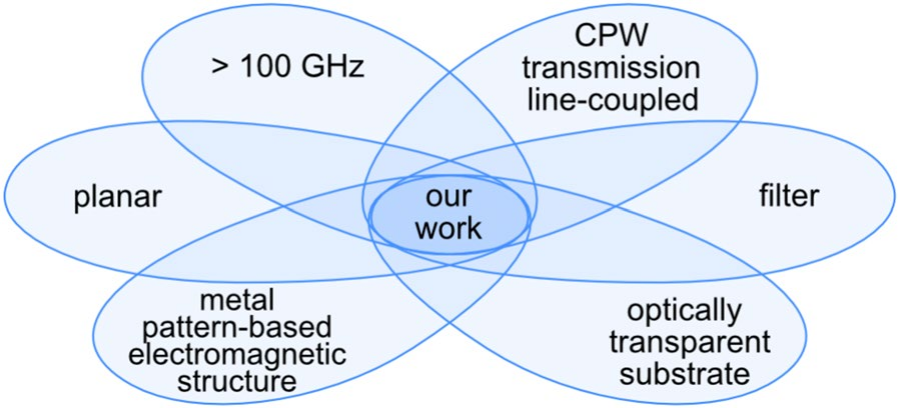
The unique characteristics and novelty our device depicted in Venn diagram positioning our work at the intersection of six key attributes not present in any other work in the available literature: planar geometry, metal-pattern-based electromagnetic structure, operation above 100 GHz, direct coplanar waveguide transmission line coupling, filter functionality, and implementation on an optically transparent substrate.

Usually, a resonator is the key geometric structure of filter designs. There is a wide variety of them, such as ring resonator^35^, split ring resonator (SRR)^36^, complementary SRR^37–39^ spiral resonator^40–42^, $\lambda$/4 open-end stub resonator^43–48^, and various types of defected ground structures^49–51^. A resonator coupled to a coplanar waveguide (CPW) can also be used as a sensor^52^. Spoof surface plasmon polaritons can also be employed as a structure for a CPW filter^53,54^. Lots of these designs are bulky and cover a substantial part of the CPW’s ground plane. Another problem is that the device topologies often contain diminutive patterns, which work well at frequencies up to around 10 GHz but cannot be easily scaled above 100 GHz^53^. Sometimes, additional manufacturing processes such as air bridges^45,47^ or usage of membrane-supported CPW^55^ are needed. Overall, the unique niche of our device is summarized in Fig. 1. The only conceptually related device^56^ (> 100 GHz planar, metal pattern-based, on a transparent substrate, filter, transmission line-coupled) we found in the literature is a band-pass filter, not a band stop-filter, operating on different frequency band (140 - 190 GHz) than our and coupled to a Goubau line, which is challenging to integrate with the common circuit structures, which are typically based on coplanar waveguide topology.

**Fig. 2:**
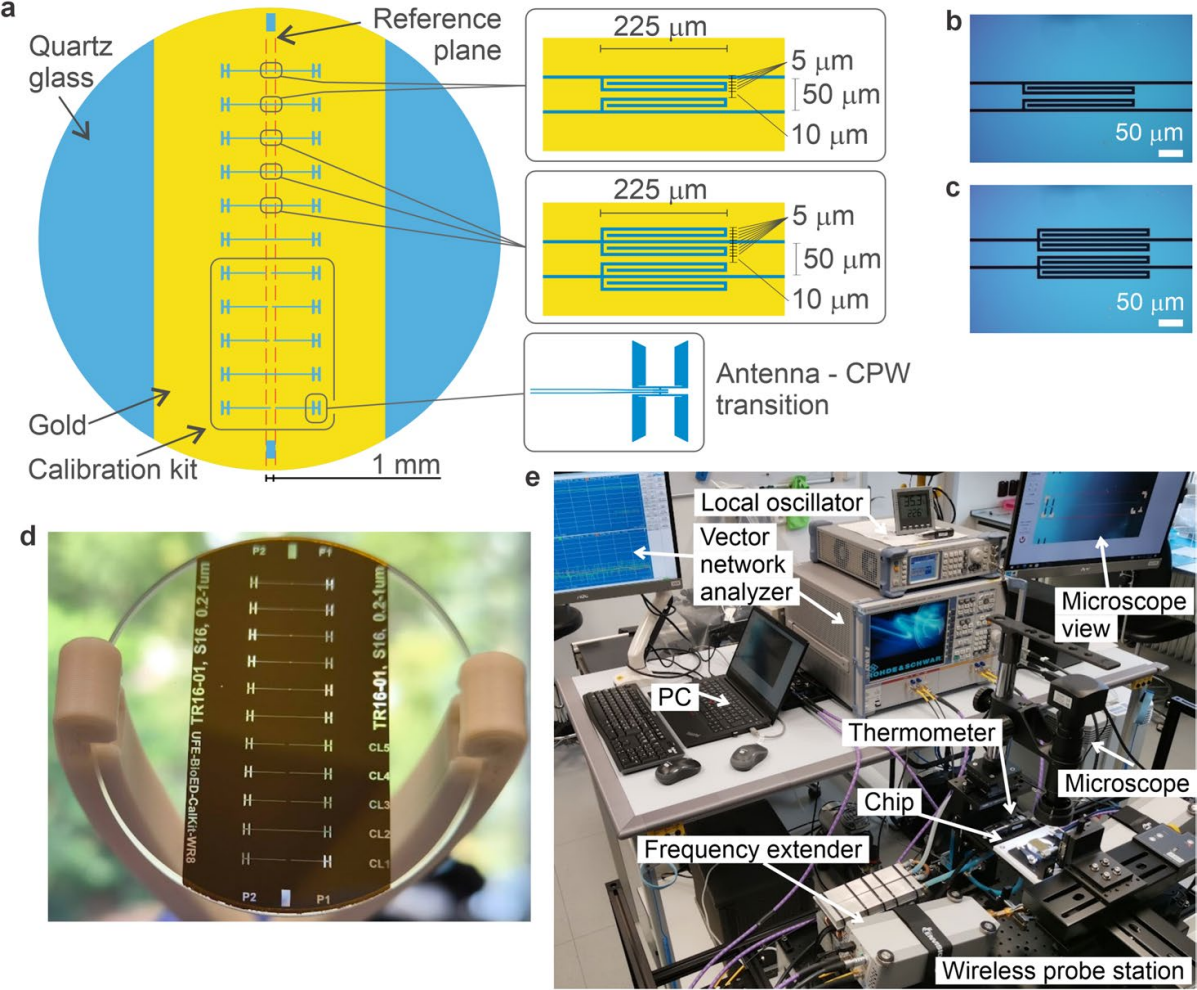
The design of the 2-inch diameter round chip with the filter units and experimental measurement setup. (**a**) The calibration kit contains five pairs of shift shorts and one thru line based on a coplanar waveguide transmission line on the quartz glass substrate in the bottom half of the chip. The two types of filter units (3x strip + ground pairs, 2x strip pair) and one thru line are on the top half of the chip. Zoom on the units situated in the middle of the CPW lines. (**b**) The fabricated chip fixed in the tweezer. (**c**) and (**d**) A microscopic image (Nomarski microscopy) of the fabricated filter units. (**e**) The experimental setup contains VNA-ZVA67, frequency extenders, and a TeraProbes wireless probe station.

The design proposed in this paper features open stub geometry, which is easy to manufacture and does not occupy the CPW’s ground plane at all in the variant with the best performance; see Fig. 2b. Such a design is suitable for mass-produced low-cost either powered by energy harvesting^57^ or fully passive devices, which are expected in both 5G and the upcoming 6G standard.

In this paper, we show the design and fabrication of the band-stop filter unit within the F-band (90-140 GHz) embedded in a CPW. The unit is based on the $\lambda$/4 open-end stub resonator, which is folded in the middle. This resonator can be coupled with CPW’s ground plane or center strip; see Fig. 2 or both; see Fig. 2c. All variants were analyzed and could be cascaded into a higher-order filter analogously to^35,45,46,48,51,55^. The center band-stop frequency ${f_{0}}$ was set to 115 GHz, but it can be easily changed by adjusting the length of the folded open-end stub. The filter unit was designed and simulated using CST Studio Suite$^{\tiny \circledR }$ and fabricated on a quartz glass substrate using direct optical lithography.

## Results and discussion

### The folded stub resonator design principles

The topology of the proposed band-stop filter unit is based on an open-end stub resonator, which is folded in the middle. The stub functions as a CPW short when its length equals the $\lambda$/4 of the transmitted signal. The first variant consists of four identical resonators that are symmetrically coupled to a CPW in order to form a filter unit with higher insertion loss. Two resonators are coupled to the center strip (strip pair) and two to the ground plane (ground pair); see Fig. 2c. The second variant consists only of the strip pair; see Fig. 2b. The symmetry of the filter unit geometry prevents the propagation of parasitic coupled slot line mode in the CPW. Therefore, the designed filter does not require any air bridge, which is a substantial advantage from a manufacturing process point of view^58^.

The proposed filter unit was simulated and manufactured on an optically transparent quartz glass substrate with height ${h_{1}}$ = 3.2 mm, a relative dielectric constant ${\epsilon _{r1}} = 3.8$, and a loss tangent of 0.01. The metal pattern is 300 nm thick gold layer with a conductivity of 45.6 MS/m, physical vapor-deposited on the top of the substrate. The ability to optically access the CPW gaps is crucial for potential spectroscopic and microscopic applications, as the field strength is maximized in the regions between the ground and the central conductor.

See a detailed description of the manufacturing process described in the Methods section. The filter unit’s frequency ${f_{0}}$ was set to 115 GHz. The length of the inner edge of the folded stub ${l_{stub}}$ is equal to $\lambda$/4 according to the frequency ${f_{0}}$; see equation (1), where c_0_ is speed of light.

1 $$\begin{aligned} l_{stub}=\frac{{c}_{0}}{4{f}_{0}\sqrt{{\varepsilon }_{eff}}} \end{aligned}$$

Effective permittivity ${\varepsilon }_{eff}$ can be generally obtained using a complete elliptic integral of the first kind; see equations (2) and (3)^59^. One of the dielectrics is air in our case. Therefore ${\epsilon _{r2}}$ = 1, which removes the second term in the brackets (2). The desired frequency ${f_{0}}$ is achieved by ${l_{stub}}$ = 430 $\upmu$m according to this method. It can also be easily tuned to higher frequencies just by reducing the length of the stub. The cutoff frequency of the current CPW without any resonators is approximately 160 GHz as a consequence of high radiation losses.

2 $$\begin{aligned} \varepsilon _{eff}=1+\frac{1}{2}\frac{\textrm{K}\left( k \right) }{\textrm{K}\left( {k}' \right) }\left[ \left( \varepsilon _{r1}-1 \right) \frac{\textrm{K}\left( {k}'_{1} \right) }{\textrm{K}\left( k_{1} \right) }+\left( \varepsilon _{r2}-1 \right) \frac{\textrm{K}\left( {k}'_{2} \right) }{\textrm{K}\left( k_{2} \right) }\right] \end{aligned}$$

where

3 $$\begin{aligned} k_{1,2} = \sqrt{1-\left( {k}'_{1,2} \right) ^{2}}, \;\;\;\quad {k}'_{1,2}=\frac{\sinh \left( \pi s/ (4h_{1,2} ) \right) }{\sinh \left( \pi \left( s+2w \right) / (4h_{1,2}) \right) } \end{aligned}$$

The width of the CPW strip is 50 $\upmu$m, and the width of the gap between the strip and the ground is 5 $\upmu$m. The resonator line width and the width of gaps between resonators and the CPW is also 5 $\upmu$m; see Fig. 2a. The single folded stub is 225 $\upmu$m by 20 $\upmu$m (gaps included). The filter unit variant consisting of four resonators has a compact footprint of 0.023 $\hbox {mm}^2$. The variant using only strip resonator pair has only a 0.011 $\hbox {mm}^2$ footprint; see Fig. 2c and d respectively.

### The filter unit performance

**Fig. 3:**
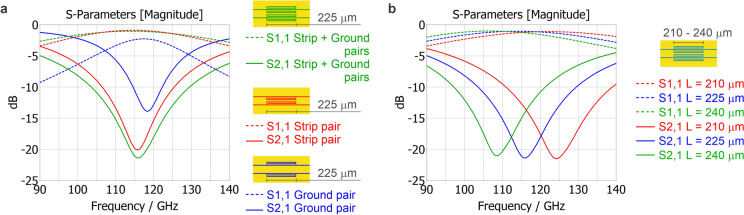
(**A**) The simulated S-parameters of three variants of the filter unit using a folded stub resonator coupled to a CPW. S11 and S21 magnitudes are depicted. (**B**) The simulated S-parameters compare three designs using the same arrangement of four folded stub resonators, which differ only in their length. S11 and S21 magnitudes are depicted.

The basic building block of the filter unit is the $\lambda$/4 folded open-end stub resonator. There are two pairs of identical resonators symmetrically coupled to a CPW. The first pair is coupled to the center strip and the second pair to the ground plane; see Fig. 2a. Frequency domain simulations were performed for a complete filter unit containing both resonator pairs and also for each pair separately on a 1 mm long CPW line, which itself has an insertion loss of 0.8 dB at the frequency ${f_{0}}~=~115$ GHz. The ground pair itself has maximal insertion loss in the stop band of only 13.9 dB, the strip pair itself has 20.1 dB, and all four stubs (both pairs together) have 21.4 dB according to simulations; see Fig. 3a. These results suggest that the strip pair is dominant and can be used as a stand-alone filter unit. The dependence of the frequency ${f_{0}}$ on the length of the folded stubs is depicted in Fig. 3b. Approximately two micrometers per gigahertz are needed to change the frequency ${f_{0}}$. All simulation data were obtained using CST Studio Suite$^{\tiny \circledR }$.

**Fig. 4:**
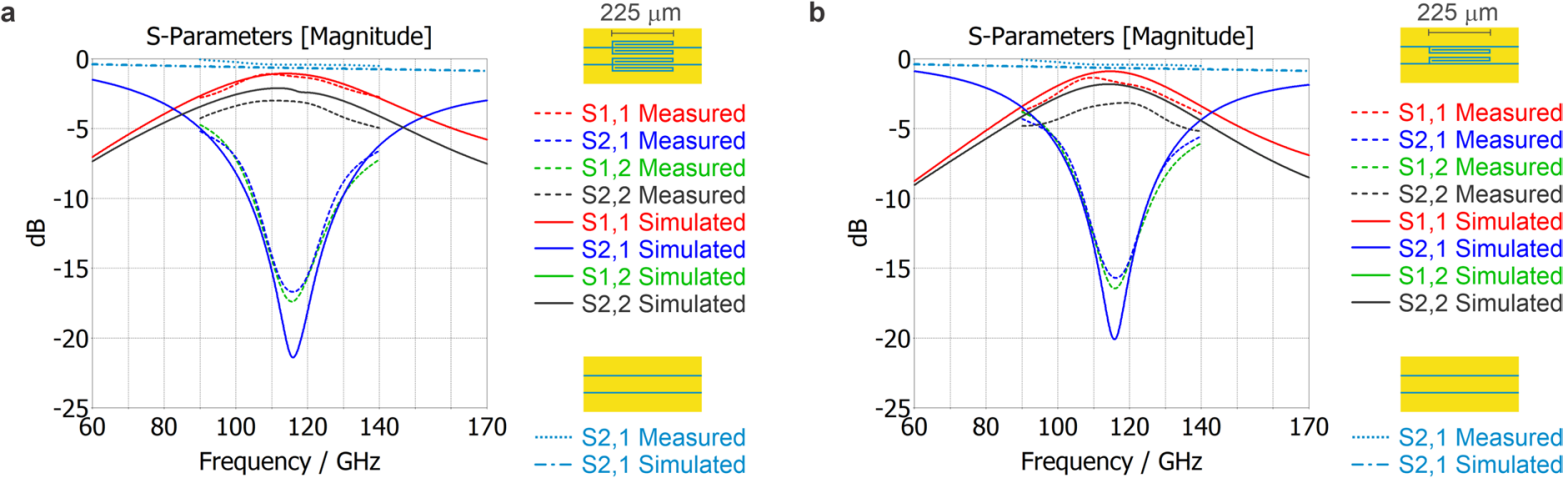
Comparison of measured and simulated data. S11 and S21 magnitudes are depicted. (**a**) The variant with both strip and ground pairs. (**b**) The strip pair only variant. For reference, the performance of a bare coplanar transmission line is also included in the plots.

To demonstrate the filter unit functionality, we fabricated the devices by optical lithography, see Methods. Then we obtained the S-parameters of the device from experimental measurement and compared them to the simulations. We found there is a negligible difference of less than 0.5 GHz in the frequency ${f_{0}}$ between measured and simulated S-parameters. The measured maximal insertion loss at frequency ${f_{0}}$ is lower than the simulated one by 4.7 dB and 4.4 dB in the case of the four stubs filter unit variant and the strip pair, respectively. According to measurement, the filter unit containing all four stubs has a 6 dB bandwidth of 39.4 GHz (34.3%); see Fig. 4a. The strip pair variant has a 6 dB bandwidth of 30.0 GHz (26.1%); see Fig. 4b. For details regarding the phase, please refer to the Supplementary Information, SI-1 Group delay.

To quantitatively assess the filter performance we define the skirt factor

$$\begin{aligned} SF=\frac{f_{\,|S_{21}|=-15\;\textrm{dB}}}{f_{\,|S_{21}|=-3.5\;\textrm{dB}}}. \end{aligned}$$

In this definition, we employ $-3.5\;\textrm{dB}$ (power transmission $=44.7\%$) rather than the conventional $-3\;\textrm{dB}$ (50%) because the available measurement band does not extend to the frequency where $|S_{21}|=-3\;\textrm{dB}$. For the stop-band onset we use $-15\;\textrm{dB}$ (power transmission $=3.1\%$) instead of the typical $-20\;\textrm{dB}$ (1%), as a single filter section does not reach $-20\;\textrm{dB}$ rejection. With this definition the measured response (Fig. 4b) yields $SF \approx 1.35$, while the simulated response gives $SF \approx 1.25$, indicating a relatively soft transition. If a sharper skirt is required, cascading units is effective: three sections in series reduce the skirt factor to $SF \approx 1.14$ (see Fig. SI-2).

The filter lends itself to a variety of applications. The use of broadband, mild-attenuation filters might be beneficial in situations where a signal with a number of high-power spectral peaks needs to be gently suppressed without a significant distortion of the pulse’s time-domain shape. For example, a periodic Dirac pulse, which is generally used for sampling, features a periodic needle spectrum in the sub-terahertz band, which can spread in a device and harm the function of its other parts. This can also contradict EMI (Electromagnetic Interference) compliance. EM emissions are typically measured from 9 kHz to 18 GHz, but CISPR 11 regulation applies to industrial, scientific, and medical electrical equipment operating up to 400 GHz^60^. Another potential specific application of the filter is to work with the 118 GHz molecular oxygen line. Atmospheric $\hbox {O}_2$ is an emitter^61,62^ and absorber^63^ at this frequency. Ground-based sub-THz instruments (such as telescopes that monitor the cosmic microwave background) must attenuate that $\hbox {O}_2$ emission line before it reaches the low-noise amplifier to prevent receiver saturation. Conversely, active $\hbox {O}_2$-sounding radars transmit powerful 118 GHz pulses^64–66^ other equipment working nearby can insert the same band-stop filter at its input to reject this interference.

## Conclusion

This paper presents the design, fabrication, and measurement of a novel band-stop filter unit operating in the F-band (90–140 GHz), implemented on an optically transparent quartz glass substrate. The device’s unique design, based on a compact $\lambda$/4 open-end folded stub resonator, offers strong features, including compatibility with advanced optical microscopy techniques, compactness, and manufacturability. Among the analyzed variants, the design utilizing only the strip resonator pair proved to be the most promising due to its minimal footprint, occupying only $\lambda$/8 of the coplanar waveguide, while maintaining high performance with a slightly narrower bandwidth compared to the four-stub variant.

Future work will focus on optimizing the device’s performance currently constrained by metal and dielectric losses, refining the design for higher-order filtering, and integrating the device with microfluidic systems for advanced biophysical and spectroscopic applications. This filter unit design, characterized by its small size, scalability, and ease of production, is well-suited for the 90-140 GHz frequency bands which overlaps with the bands of the emerging 5G/6G technologies and holds potential for specialized applications.

## Methods

### Electromagnetic simulation

Electromagnetic simulations were obtained using full-wave Frequency Domain Solver embedded into CST Studio Suite$^{\tiny \circledR }$ software. The geometric structure of the filter was defined in the first step. Then it was divided into tetrahedral elements (3D mesh), with an initial size significantly smaller than the resonant wavelength of the structure. Automatic Adaptive Mesh Refinement was applied after. S-parameters and field plots were obtained by embedded finite element method (FEM). See the.cst CST Studio Suite raw files for all data presented in this paper in https://doi.org/10.5281/zenodo.14055224. Detailed analysis led to the optimization of the structure for the best achievable results.

### Fabrication

The chip was fabricated using standard microtechnological procedures combined with maskless direct-write optical lithography. A round quartz glass substrate (2-inch diameter, 0.125-inch thickness) was sourced from Technical Glass Products, Inc. Initial cleaning involved sequential spraying with acetone, deionized water, and isopropyl alcohol, followed by oxygen plasma treatment for 4 minutes. The substrate was then dehydrated by heating on a hotplate at 200 $^\circ$C for 15 minutes, followed by a 4-minute cooling period. Two photoresist layers were spin-coated: a 0.4 $\upmu$m layer of LOR 5a and a 1.3 $\upmu$m layer of S1813 G2. The sample was soft-baked at 120 $^\circ$C for 155 seconds on a hotplate. Patterning was carried out using a MicroWriter ML2 system operating at a wavelength of 405 nm. Following exposure, a post-bake step was performed at 120 $^\circ$C for 1 minute on a hotplate, followed by a 2-minute cooling period. The resist was developed in MF-319 developer for 95 seconds with gentle agitation. The substrate was then rinsed with deionized water, treated with UVO-Cleaner (Model 30, Jelight Co. Inc.) for 10 minutes, and exposed to oxygen plasma (Nano, Diener electronic GmbH & Co KG) for 1 min with 30 % power. Metallization was achieved by evaporating a 10 nm titanium adhesion layer, followed by deposition of 300 nm of gold by electron-beam evaporation (PLS570 PMK01-466, Pfeiffer Vacuum). Finally, the lift-off process was performed using mr-Rem 700 remover, after which the sample was rinsed with isopropyl alcohol and dried with nitrogen gas.

### Measurement setup and calibration

The measurement setup (see Fig. 2e) consisted of a two-port vector network analyzer (VNA; R&S$^{\circledR }$ ZVA67, Rohde & Schwarz$^{\circledR }$), a signal generator (SMB100A, Rohde & Schwarz$^{\circledR }$) used as a local oscillator, and two frequency extenders (ZC140, Rohde & Schwarz$^{\circledR }$) operating in the 90–140 GHz frequency band. An automated wireless probe station (TP-100-A8025, TeraProbes Inc.) was used for non-contact antenna-based probing. The VNA settings were configured to 501 linear frequency points, 1 kHz resolution bandwidth (RBW), and time-domain gating centered at 10 ps with a span of 100 ps.

Prior to the measurement, a non-contact on-chip calibration procedure was carried out to define the measurement reference planes at the chip surface. This calibration involved recording 2-port *S*-parameter measurements of five offset-short standards and one Through standard, all fabricated on the lower part of the same chip. The chip was automatically aligned under a fused quartz lens using a computerized micro-positioning system and a digital microscope, ensuring highly repeatable antenna alignment with sub-micrometer precision.

After the calibration standards were measured and the VNA calibration was activated, the subsequent DUT *S*-parameter measurements were automatically referenced to the on-chip reference planes, as illustrated in Fig. 2a. Since both the calibration standards and the DUT share identical on-chip antenna designs and are measured under the same optical alignment conditions, the procedure eliminates the need for physical contact during both calibration and measurement. This significantly reduces the risk of mechanical damage and allows for reliable and repeatable high-frequency characterization. Finally, *S*-parameters of the DUTs were measured.

## Supplementary Information

Below is the link to the electronic supplementary material.

Supplementary Material 1

## Data Availability

Raw data are available in the Zenodo database under https://doi.org/10.5281/zenodo.14055225.
