# Supplementary material for: Metal pattern-based planar sub-THz filter in coplanar waveguide on optically transparent substrate
Source: Sci Rep. 2025 Sep 26;15:33167. doi: 10.1038/s41598-025-15178-3 (PMC12475208; doi:10.1038/s41598-025-15178-3)
Supplement: Supplementary file 1 — Supplementary Material 1 [file 41598_2025_15178_MOESM1_ESM.pdf]

Supplementary Information

Metal Pattern-Based Planar Sub-THz Filter in Coplanar Waveguide on Optically Transparent Substrate

Jaroslav Havlíček, Daniel Havelka, and Michal Cifra

SI-1 Group delay

Figure SI-1 presents the group delay characteristics for the two waveguide variants. Both measured and simulated data are shown for the S21 and S12 transmission parameters across the frequency range from 90 to 140 GHz.

In panel a, the structure includes both the strip and ground conductor pairs, while panel b corresponds to the configuration with the strip pair only. A pronounced dip in group delay is observed around 117 GHz in both cases, corresponding to the frequency region of maximum coupling. The simulations show good agreement with the measured data, capturing the overall shape and the peak values of the delay, although minor discrepancies are present near the resonance minimum.

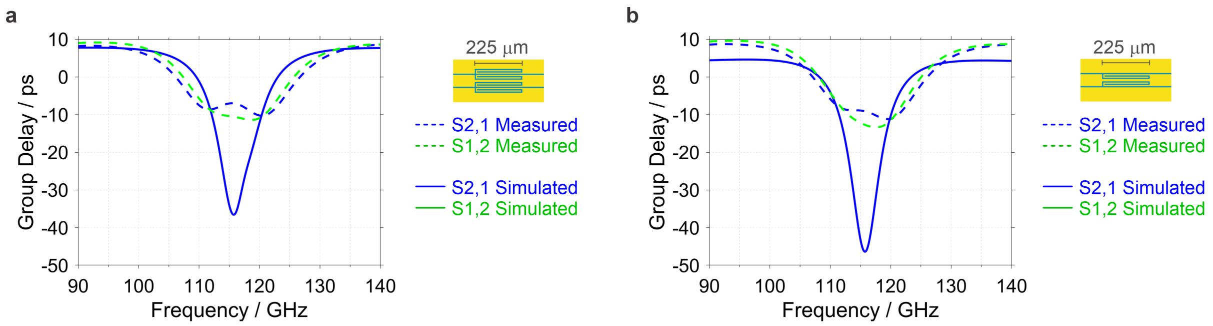

Figure SI-1: Comparison of measured and simulated group delay. S21 and S12 group delays are shown. a) The variant with both strip and ground pairs. b) The strip pair only variant.

SI-2 Effect of Multiple Resonator Sections on Transmission Characteristics

Figure SI-2 presents simulated S-parameters (S21) for structures incorporating one, two, and three resonator sections cascaded with a 90 μm gap between each section. Each resonator section contains the same number of strip and ground conductor pairs as previously analyzed.

The results show that increasing the number of cascaded sections leads to a deeper and broader attenuation band, while the frequency of the transmission minimum remains approximately constant around 117 GHz. This behavior is consistent with stronger coupling and the presence of multiple resonances within the structure. Such characteristics can be advantageous in applications requiring selective filtering or engineered delay in the sub-THz frequency range.

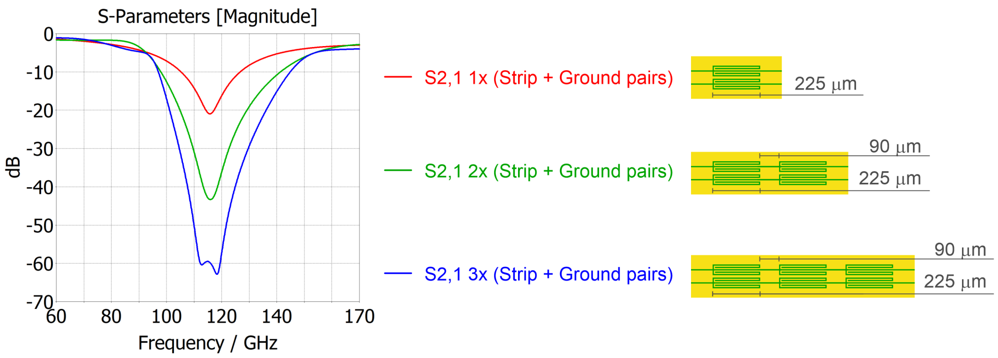

Figure SI-2: Simulated transmission parameters (S21) for structures with one (red), two (green), and three (blue) cascaded resonator sections. Each configuration includes both strip and ground conductor pairs. As the number of sections increases, the attenuation and the bandwidth broaden. The spacing between adjacent sections is 90 µm.
